# Musculoskeletal symptoms in systemic lupus erythematosus patients and their impact on health-related quality of life

**DOI:** 10.1186/s12891-024-07367-4

**Published:** 2024-04-08

**Authors:** Samar Tharwat, Sara Mahmood Husain

**Affiliations:** 1 Rheumatology & Immunology Unit, Department of Internal Medicine, Faculty of Medicine, Mansoura University, El Gomhouria St, Mansoura, Dakahlia Governorate Egypt; 2 Department of Internal Medicine, Faculty of Medicine, Horus University, New Damietta, Egypt; 3 Mansoura Manchester Programme for Medical Education, Faculty of Medicine, Mansoura University, Mansoura, Egypt

**Keywords:** Musculoskeletal pain, Systemic lupus erythematosus, Health-related quality of life, Nordic questionnaire

## Abstract

**Objectives:**

To evaluate the musculoskeletal (MSK) symptoms experienced by SLE patients and determine how those symptoms relate to their health-related quality of life (HRQoL).

**Materials and methods:**

This is a cross-sectional study that was carried out on 103 adult SLE patients. sociodemographic, clinical, and therapeutic data were recruited. They were asked to complete the following: Nordic Musculoskeletal, Short-Form McGill Pain, and Lupus QoL Questionnaires.

**Results:**

The mean age was 30.81 ± 9.44 years. There was a total of 86 females and 17 males (F: M:5:1). Almost all the patients reported MSK symptoms (96.1%). The maximum number of patients reported pain in the right and left wrist and hand (64.1%, 63.1%, respectively). One-fourth (25.2%) described at least five bodily sites of MSK symptoms, while 70.9% had more than five sites of MSK symptoms. Most of the patients described the pain as discomforting (40.8%). Patients with MSK symptoms scored significantly worse in all domains. In addition, the QoL scores of patients with more than 5 body sites of MSK symptoms were significantly lower than those of patients with fewer than 5 sites of MSK symptoms.

**Conclusion:**

SLE patients have a high MSK burden, and MSK symptoms have a negative impact on HRQoL in these patients.

## Introduction

Systemic lupus erythematosus (SLE) is a heterogenous autoimmune disease with a variable clinical course and prognosis [1]. This heterogeneity poses enormous challenges to diagnostic, medical, and therapeutic progress [2]. SLE has a remarkable female predominance, with nearly 10 female patients for each male patient. The incidence ranges from 0.3 to 31.5 cases per 100,000 people per year [3]. Although it is present in all ethnicities, it is more prevalent in non-Caucasians. SLE is uncommon in Africa, despite the fact that the prevalence of the disease is higher among people of African heritage in Europe and the United States [4, 5].

The manifestations of SLE are linked to the presence of numerous autoantibodies (Ab), which are responsible for the development and accumulation of immune complexes (ICs), in addition to other immunological processes [6]. Constitutional, mucocutaneous, and musculoskeletal (MSK) symptoms are the earliest and most commonly reported complaints among SLE patients. Nevertheless, this disease can have an impact on any organ, including the skin, hematologic, renal, neuropsychiatric (NP), cardiovascular, and/or pulmonary systems [1].

MSK symptoms, such as arthritis and arthralgia, are frequent SLE manifestations. Over the course of the disease, 53–95% of patients report MSK manifestations and joint involvement, and up to 60% during disease flares [7, 8]. SLE has a substantial impact on health related quality of life (HRQoL) [9]. SLE-related involvement in the MSK system may have a major impact on HRQoL [10].

In spite of the high frequency of MSK manifestations, there are several aspects of these manifestations that need further clarification. There have only been a few studies that have investigated the impact of MSK symptoms on HRQoL in SLE patients.

So, our aim of this study was to evaluate the MSK symptoms experienced by SLE patients and determine how those symptoms relate to their HRQoL.

## Patients and methods

### Study design and setting

This is a cross-sectional study that was conducted between April and September 2022 at the Rheumatology and Immunology Unit (inpatient and outpatient) in the Mansoura University Hospital in Egypt, which is an urban tertiary hospital. Patients who were diagnosed with SLE were included in the study in a sequential manner. All patients met the systemic lupus international collaborating clinics classification criteria [11] or the new 2019 EULAR/ACR Classification Criteria for SLE [12]. No selection process resulted in the collection of more severe cases. Individuals who were less than 18 years old, had a history of malignancy, or suffered from any other chronic rheumatic, musculoskeletal, or neurological disorder were not allowed to participate in the study from the very beginning. Each researcher held an organized in-person encounter with each patient, verbally presenting the items using simple and clear language. This approach enables more complex issues to be explored than the self-administered style and provides more detailed explanations of the queries.

### Ethical consideration

This study was conducted in accordance with the principles outlined in the Helsinki Declaration [13], and the Institutional Research Board of the Faculty of Medicine at Mansoura University provided its approval (Approval No: R.23.04.2145) to the study protocol before it was carried out.

### Sample size calculation

This calculation of the sample size was carried out using G*Power. The outcome of interest was determined to be the prevalence of MSK disorders among SLE patients, and it was approximately 85% over the course of the disease [7]. The effect size was calculated to be 0.1, the alpha error was calculated to be 0.05, and the power of the study was 0.9. Hence, the total number of individuals in the sample was determined to be 93.

### Questionnaire structure

The questionnaire included already validated questionnaires and other questions about the sociodemographic, clinical, and therapeutic data. A “yes or no” response was provided to most questions, and most of them were close ended. The questions were crafted to be straightforward and without any misinterpretation. The participants were informed that their participation in the survey is fully voluntary, and they were also given the option to decline participation. All personal information, including their names and contact information, will be kept strictly confidential and used solely for scientific study. By signing the consent form, they stated that they were willing to participate in the study.

### Sociodemographic data

The following sociodemographic data were obtained from the participants: age, gender, material status, education level, employment, residency, and socioeconomic status.

### Clinical data of lupus

Participants were questioned on their age when they received their initial diagnosis of lupus, the length of time they had been living with the disease, and whether or not they had a previous history of renal transplantation, nephritis, dialysis, seizures, strokes, or psychiatric diagnoses. Then, clinical evaluation was done, and the SLE disease activity index (SLEDAI) [14] was assessed. On the basis of SLEDAI scores, the following activity categories have been defined: no activity (SLEDAI = 0), mild activity (SLEDAI = 1 to 5), moderate activity (SLEDAI = 6 to 10), high activity (SLEDAI = 11 to 19), and very high activity (SLEDAI ≥ 20) [15].

### SLE therapeutic data

Therapeutic data were obtained from the participants. They were questioned regarding the medications that they take for the management of their condition, such as corticosteroids, antimalarials, azathioprine, methotrexate, mycophenolate, or any other immunosuppressive drugs or biologics.

### Musculoskeletal discomfort form

The Nordic Musculoskeletal Questionnaire (NMQ-E), which is valid and reliable, was essentially used to assess the existence and distribution of MSK symptoms such as ache, pain, discomfort, and numbness carefully at different body areas. The NMQ-E questionnaire comes with a picture that the patient can look at and pinpoint the approximate location of the portions of the body that he is having trouble with [16].

### Short-form McGill pain questionnaire (SF-MPQ)

It is a valid and reliable evaluation that is quicker and simpler to utilize in clinical research; it was used to measure the pain sensory intensity [17]. The Arabic version of the SF-MPQ was utilized for this research because it has been shown to be reliable and valid among Arabic-speaking patients [18]. It included 15 descriptors, four of which were affective and eleven of which were sensory. Each descriptor was graded on a scale from 0 (none) to 1 (mild) to 2 (moderate) to 3 (severe) on an intensity scale. The Present Pain Intensity (PPI) index of the standard McGill Pain Questionnaire (MPQ) and a Visual Analogue Scale (VAS) were both incorporated in the Short Form McGill Pain Questionnaire (SF-MPQ) in order to assess the experience of pain intensity [19]. The total score on the questionnaire ranged from 0 to 45 on the Pain Rating Index (PRI), including (Affective Subscore: 0/12 & Sensory Subscore: 0/33), from 0 to 5 on the PPI, and from 0 to 10 centimeters on the VAS [20].

### Lupus quality of life (Lupus QoL)

This is the most commonly studied SLE-specific HRQoL score, developed and validated in SLE adults from the United Kingdom [21]. It is comprised of 34 questions derived from SLE patients and organized into eight domains, including physical health, emotional health, body image, pain, planning, fatigue, intimate relationships, and burden on others. The questions were based on the patient’s experience in the previous four weeks, and responses were given on a 5-point Likert scale (0–4, where 0 means always and 4 means never). A summary Lupus QoL score of 0 to 100 was presented, with higher values indicating better HRQoL, 0 representing the worst HRQoL, and 100 representing the best HRQoL. The respondent took less than 10 min to complete.

### Statistical analysis

In order to do analysis on the collected data, the Statistical Package for Social Science (SPSS) version 22 program was utilized. When presenting quantitative data, we used means and standard deviations (SD) for parametric variables and median (min-max) for nonparametric variables. When presenting qualitative data, we used percentages and numbers. The Shapiro-Wilk test was carried out in order to ascertain whether or not the variable distribution was normal. The Kruskal-Wallis test was utilized in order to analyze the nonparametric data.

## Results

The study comprised 103 SLE patients recruited from an Egyptian rheumatology and immunology clinic. Their mean age was 30.81 ± 9.44 years. There were a total of 86 females and 17 males (F: M:5:1). Sociodemographic characteristics are illustrated in Table 1. Among them, 59.2% were married, 62.1% had graduated from college, 58.3% originated from urban areas, and 80.6% had a socioeconomic standing that was moderate. Only 13.6% of participants were current smokers, while 22.3% of participants exercised regularly.

**Table 1:** Sociodemographic data of the studied SLE patients (*n* = 103)

Variable mean ± SD, n (%)	Total SLE patients (*n* = 103)
Age (years)	30.81 ± 9.44
Sex	
Male Female	17 (16.5) 86 (83.5)
Marital status	
Single Married Widow Divorced	36 (35.0) 61 (59.2) 3 (2.9) 3 (2.9)
Living alone	10 (9.7)
Education level	
Not educated Primary school Secondary school High school Graduate Postgraduate	3 (2.9) 3 (2.9) 7 (6.8) 23 (22.3) 64 (62.1) 3 (2.9)
Residence	
Rural Urban	43 (41.7) 60 (58.3)
Employment status	
Not employed Employed Retired Student	47 (45.6) 40 (38.8) 2 (1.9) 14 (13.6
Socioeconomic status	
Low Moderate High	15 (14.6) 83 (80.6) 5 (4.9)
Life habits	
Smoking Exercise practice	14 (13.6) 23 (22.3)
Treatment payment system	
Health insurance State expense Patient expense	10 (9.7) 61 (59.2) 32 (31.1)

Clinical and therapeutic data of the participants are illustrated in Table 2. The mean age at lupus diagnosis was 24.82 ± 8.49 years, with a median disease duration of 4 years. Only 11.7% had biopsy-proven nephritis, and 2.9% were on maintenance hemodialysis. According to SLEDAI, 35.9% had mild disease activity, whereas 37.9% had moderate disease activity. treatment received in the following descending frequency: corticosteroids (80.6%), antimalarials (54.4%), azathioprine (52.4%), mycophenolate mofetil (22.3%), methotrexate (20.4%), and biologics (8.7%).

**Table 2:** Clinical and therapeutic data of the study SLE patients (*n* = 103)

Variable mean ± SD, n (%), median (min-max)	Total SLE patients (*n* = 103)
Age at diagnosis (years)	24.82 ± 8.49
Diseases duration (years)	4 (1–39)
Clinical manifestations	
Psychiatric diagnosis	11 (10.7)
Seizures	11 (10.7)
Stroke	6 (5.8)
Biopsy proven nephritis	12 (11.7)
Dialysis	3 (2.9)
Disease activity (SLEDAI)	
No Mild Moderate Sever	19 (18.4) 37 (35.9) 39 (37.9) 8 (7.8)
SLE medication classes	
Steroids	83 (80.6)
Antimalarials	56 (54.4)
Azathioprine	54 (52.4)
Mycophenolate Mofetil	23 (22.3)
Methotrexate	21 (20.4)
Biologics	9 (8.7)

SLEDAI: systemic lupus erythematosus diseases activity index

**Fig. 1:**
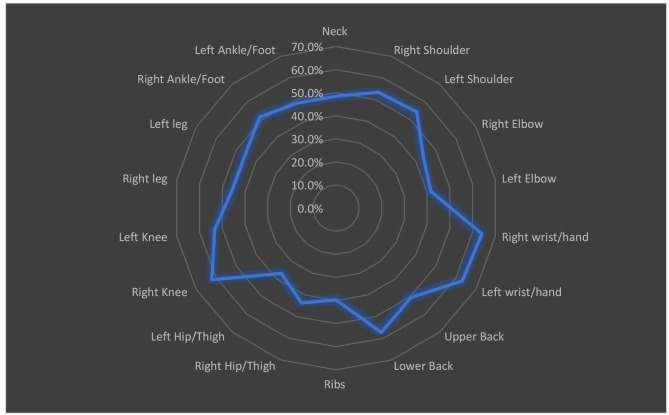
MSK symptoms among the studied SLE patients (*n* = 103)

The frequency of MSK symptoms (ache, pain, discomfort, and numbness) in the study SLE patients is shown in Fig. 1. According to the body regions, the maximum number of patients reported pain in the right and left wrist and hand (64.1%, 63.1%, respectively), followed by right knee (62.1%) and lower back (57.3%), followed by the left and right shoulder (54.4%, 53.4%, respectively) and left knee (53.4%). Ribs (39.8%) and left hip and thigh (36.9%) were the least common symptoms.

According to the Nordic MSK Questionnaire, almost all the studied SLE patients reported MSK symptoms (96.1%). As indicated in Table 3, approximately one-fourth (25.2%) described at least five bodily sites of MSK symptoms, while 70.9% had more than five sites of MSK symptoms. The length of time that patients experienced MSK symptoms varied, with 49.5% reporting MSK symptoms for more than three months.

**Table 3:** Duration and number of MSK symptoms per individual in the study SLE patients (*n* = 103)

Variable n (%)	Total SLE patients (*n* = 103)
MSK symptoms	
No symptoms	4 (3.9)
At least 5 body sites	26 (25.2)
> 5 body sites	73 (70.9)
Duration of MSK symptoms	
< 6 weeks	27 (26.2)
6 weeks − 3 months	25 (24.3)
≥ 3 months	51 (49.5)

The SF-MPQ scores of the participants in the study are outlined in Table 4. Most of the patients described the pain as discomforting (40.8%), while only 3.9% described it as excruciating. The pattern of pain is predominantly intermittent (61.2%), whereas it is continuous in only 13.6% of cases. The median value of VAS was 6.

**Table 4:** Short-Form McGill Pain score in the study SLE patients (*n* = 103)

Variable mean ± SD, n (%),	Total SLE patients (*n* = 103)
Sensory component	13 (0–30)
Affective component	5 (0–12)
Total Descriptor	19 (0–42)
Present Pain Intensity (PPI) index	
No pain	4 (3.9)
Mild	20 (19.4)
Discomforting	42 (40.8)
Distressing	16 (15.5)
Horrible	17 (16.5)
Excruciating	4 (3.9)
Pattern of pain	
Repeated after a short period of time	22 (21.4)
Intermittent	63 (61.2)
Continuous	14 (13.6)
Visual analogue scale	6 (0–10)

The study employed the scores of lupus QOL domains according to the distribution of MSK symptoms. As indicated in Table 5, patients with MSK symptoms scored significantly worse in all domains than those without symptoms. In addition, the QoL scores of patients with more than five body sites of MSK symptoms were significantly lower than those of patients with fewer than five sites of MSK symptoms.

**Table 5:** Distribution of Lupus QOL domains according to the distribution MSK symptoms in the study SLE patients (*n* = 103)

Variable	Total	Musculoskeletal symptoms			P
		No *n* = 4	≤ 5 discomforts *n* = 26	> 5 discomforts *n* = 73	
Physical health	43.75 (0-100)	100 (87.50–100	50 (6.25–100)	37.5 (0-100)	0.001*
Pain	50 (0-100)	100 (100–100)	58.33 (0-100)	41.67 (0-100)	0.001*
Planning	50 (0-100)	100 (100–100)	50 (0-100)	50 (0-100)	0.002*
Intimate relationships	75 (0-100)	100 (100–100)	75 (0-100)	50 (0-100)	0.002*
Burden to others	50 (0-100)	100 (100–100)	58.33 (0-100)	50 (0-100)	< 0.001*
Emotional health	45.83 (0-100)	100 (95.83–100)	56.25 (0-100)	41.67 (0-87.50)	< 0.001*
Body image	50 (0-100)	100 (75–100)	65 (5-100)	45 (0-100)	0.002*
Fatigue	43.75 (0-100)	100 (87.5–100)	50 (0-100)	37.5 (0-93.75)	0.001*

*p < 0.05

## Discussion

To the best of our knowledge, this is the first detailed study of MSK problems experienced by SLE patients that utilizes the Nordic MSK Questionnaire. The study included those diagnosed with SLE, and the results provide information regarding the link between MSK symptoms and HRQoL in these patients. The present study revealed that SLE patients have a high prevalence of MSK symptoms and that the elevated prevalence of these symptoms is also associated with a decline in their HRQoL. The findings of this study indicated that SLE patients struggle with a variety of symptoms that may influence their perception of their position in life and impede their physical, intimate, and emotional health.

In the current study, almost all of the SLE patients (96.1%) had MSK symptoms. MSK manifestations are among the most prevalent characteristics of SLE, both in terms of initial diagnosis and long-term treatment [22]. MSK manifestations of SLE can be the first symptom to appear in up to half of all SLE patients and can impact up to 95% of patients at some point during the course of the disease [8, 23]. As a result, MSK manifestations are a frequent reason for clinical trial inclusion. For example, in the phase III ILLUMINATE trial, 81% of patients had MSK activity at baseline [24]. Joint involvement in SLE is typically non-deforming and non-erosive. Jaccoud’s arthropathy (JA) is characterized by deformities in 5–15% of patients without radiographic erosions, whereas radiographic erosions may be detected in less than 5% of patients, suggesting the overlap between rheumatoid arthritis (RA) and SLE known as rhupus syndrome [25]. Comorbidities such as fibromyalgia (6–32%) [26, 27], fragility fractures (8–12%) [28, 29], and osteonecrosis (2–12%) [30] also contribute to MSK manifestations in SLE. Lupus arthritis is distinguished by a pattern of involvement that is typically, but not always, symmetric and that is localized in small joints. Because of the existence of joint effusion or synovial proliferation, the joints that are affected may appear to be swollen and/or reddened [25].

In the current study, about 71% of our cohort reported MSK symptoms in more than five body areas, and about half of them (49.5%) had MSK symptoms lasting for more than 3 months. These widespread and chronic MSK symptoms call into question the diagnosis of FM. Although FM is common in SLE patients, the condition is frequently underdiagnosed by physicians [31]. The widespread MSK pain and heightened sensitivity to pain are two of the most prominent symptoms of FM, which is a condition that can be debilitating on an emotional, social, and physical level [32]. FM can also be identified in patients with SLE, with the prevalence of the condition ranging from 8 to 61% [33, 34]. In fact, the prevalence of FM in Caucasian SLE patients is high compared to that of the general population, and it is significantly greater in patients who are in the later stages of the disease [26].

Bearing in mind that inflammatory MSK manifestations typically take the form of transient and migratory arthralgia (30–50%), fleeting arthritis (25–40%), or persistent arthritis (10–15%) [35], when we looked at the pain intensity in our cohort, we found only 19.4% with mild pain, while most of the patients described the pain as discomforting (40.8%). Several observations and reports have cast doubt on the idea that joint involvement in SLE is generally mild and only occasionally severe. This is a belief that has been questioned. One of the most frequent and incapacitating clinical manifestations that patients frequently describe is joint pain [36]. The presence of high pain scores in patients with SLE has been associated with a decline in quality of life, as well as fatigue, anxiety, and depression [37]. When viewed from a clinical standpoint, joint involvement can take place at any point throughout the course of the disease. It is distinguished by a wide variety of phenotypes and degrees of severity, ranging from mild arthralgia to erosive arthritis and causing significant functional disability [25, 38].

Despite the treatment that is currently available, individuals with SLE have a lower quality of life and a higher rate of work disability, and MSK symptoms are one of the strongest determinants of this [39, 40]. According to our findings, patients with MSK symptoms scored significantly worse in all domains than those without symptoms. In addition, the QoL scores of patients with more than 5 body sites of MSK symptoms were significantly lower than those of patients with fewer than 5 sites of MSK symptoms. According to a study conducted by Malcus Johnsson and colleagues, 73% of SLE patients struggle with hand difficulties that get in the way of their day-to-day activities [41].In addition, FM is a significant predictor of poorer self-reported quality of life in SLE patients, as has been previously demonstrated [42, 43].

This study has a number of limitations that need to be addressed. It’s possible that the results of the study were influenced by the way the data were collected, which relied mainly on self-reporting. The absence of a control group in this study is another limitation of this study. Additionally, it would be preferable if further research could identify the causes of these MSK manifestations and their predictors. Assessing the prevalence of FM in Egyptian patients with SLE would also be interesting. To evaluate the relationship between MSK manifestations and HRQoL in SLE patients, we recommend to compare them to a community-based control group.

In conclusion, the findings of this study indicate a high prevalence of MSK symptoms among SLE patients as well as a detrimental effect on HRQoL. The wrists and hands appear to be the most afflicted parts of the body. Further, longitudinal studies are required to investigate, identify, and treat MSK manifestations in SLE patients.

## Data Availability

The datasets used and/or analysed during the current study are available from the corresponding author on reasonable request.

## Abbreviations

Ab: Autoantibodies
HRQoL: Health related quality of life
ICs: Immune complexes
Lupus QoL: Lupus Quality of Life
MSK: Musculoskeletal
NMQ-E: Nordic Musculoskeletal Questionnaire
NP: Neuropsychiatric
PPI: Present Pain Intensity
SF-MPQ: Short-Form McGill Pain Questionnaire
SLE: Systemic lupus erythematosus
SLEDAI: SLE disease activity index

## References

[1] Gheita TA, Noor RA, Abualfadl E, Abousehly OS, El-Gazzar II, El Shereef RR (2021). Adult systemic lupus erythematosus in Egypt: the nation-wide spectrum of 3661 patients and world-wide standpoint. Lupus.

[2] Dörner T, Furie R (2019). Novel paradigms in systemic lupus erythematosus. Lancet Lond Engl.

[3] N AF, Dt TGB. B. Update οn the diagnosis and management of systemic lupus erythematosus. Ann Rheum Dis. 2021;80. 10.1136/annrheumdis-2020-218272 33051219

[4] Symmons DP (1995). Frequency of lupus in people of African origin. Lupus.

[5] Pons-Estel GJ, Alarcón GS, Scofield L, Reinlib L, Cooper GS (2010). Understanding the epidemiology and progression of systemic lupus erythematosus. Semin Arthritis Rheum.

[6] Pisetsky DS, Lipsky PE (2020). New insights into the role of antinuclear antibodies in systemic lupus erythematosus. Nat Rev Rheumatol.

[7] Torrente-Segarra V, Monte TCS, Corominas H (2018). Musculoskeletal involvement and ultrasonography update in systemic lupus erythematosus: new insights and review. Eur J Rheumatol.

[8] Zoma A (2004). Musculoskeletal involvement in systemic lupus erythematosus. Lupus.

[9] Etchegaray-Morales I, Méndez-Martínez S, Jiménez-Hernández C, Mendoza-Pinto C, Alonso-García NE, Montiel-Jarquín A (2017). Factors Associated with Health-Related Quality of Life in Mexican Lupus patients using the LupusQol. PLoS ONE.

[10] Natalucci F, Ceccarelli F, Cipriano E, Perricone C, Olivieri G, Pirone C (2021). Joint involvement influences quality of life in systemic lupus erythematosus patients. Lupus.

[11] Petri M, Orbai A-M, Alarcón GS, Gordon C, Merrill JT, Fortin PR (2012). Derivation and validation of the systemic Lupus International collaborating clinics classification criteria for systemic lupus erythematosus. Arthritis Rheum.

[12] Aringer M, Costenbader K, Daikh D, Brinks R, Mosca M, Ramsey-Goldman R (2019). 2019 European League Against Rheumatism/American College of Rheumatology classification criteria for systemic lupus erythematosus. Ann Rheum Dis.

[13] General Assembly of the World Medical Association (2014). World Medical Association Declaration of Helsinki: ethical principles for medical research involving human subjects. J Am Coll Dent.

[14] Gladman DD, Ibañez D, Urowitz MB (2002). Systemic lupus erythematosus disease activity index 2000. J Rheumatol.

[15] Mosca M, Merrill JT, Bombardieri S, Tsokos GC, Gordon C, Smolen JS (2007). Chapter 2 - Assessment of Disease activity in systemic Lupus Erythematosus. Systemic Lupus Erythematosus.

[16] Kuorinka I, Jonsson B, Kilbom A, Vinterberg H, Biering-Sørensen F, Andersson G (1987). Standardised nordic questionnaires for the analysis of musculoskeletal symptoms. Appl Ergon.

[17] Factorial validity of the short-. form McGill pain questionnaire (SF‐MPQ) - Wright – 2001 - European Journal of Pain - Wiley Online Library. https://onlinelibrary.wiley.com/doi/abs/10.1053/eujp.2001.0243?sid=nlm%3Apubmed. Accessed 26 Mar 2023. 10.1053/eujp.2001.0243 11558983

[18] Terkawi AS, Tsang S, Abolkhair A, Alsharif M, Alswiti M, Alsadoun A (2017). Development and validation of arabic version of the short-form McGill Pain Questionnaire. Saudi J Anaesth.

[19] Passavanti MB, Pota V, Sansone P, Aurilio C, De Nardis L, Pace MC (2017). Chronic Pelvic Pain: Assessment, evaluation, and Objectivation. Pain Res Treat.

[20] Kd W, Gj A, Dr M. Factorial validity of the short-form McGill pain questionnaire (SF-MPQ). Eur J Pain Lond Engl. 2001;5. 10.1053/eujp.2001.0243 11558983

[21] McElhone K, Abbott J, Shelmerdine J, Bruce IN, Ahmad Y, Gordon C (2007). Development and validation of a disease-specific health-related quality of life measure, the LupusQol, for adults with systemic lupus erythematosus. Arthritis Rheum.

[22] Mahmoud K, Zayat A, Vital EM (2017). Musculoskeletal manifestations of systemic lupus erythmatosus. Curr Opin Rheumatol.

[23] Nzeusseu Toukap A, Galant C, Theate I, Maudoux AL, Lories RJU, Houssiau FA (2007). Identification of distinct gene expression profiles in the synovium of patients with systemic lupus erythematosus. Arthritis Rheum.

[24] Isenberg DA, Petri M, Kalunian K, Tanaka Y, Urowitz MB, Hoffman RW (2016). Efficacy and safety of subcutaneous tabalumab in patients with systemic lupus erythematosus: results from ILLUMINATE-1, a 52-week, phase III, multicentre, randomised, double-blind, placebo-controlled study. Ann Rheum Dis.

[25] Ball EMA, Bell AL (2012). Lupus arthritis–do we have a clinically useful classification?. Rheumatol Oxf Engl.

[26] Torrente-Segarra V, Salman-Monte TC, Rúa-Figueroa Í, Pérez-Vicente S, López-Longo FJ, Galindo-Izquierdo M (2016). Fibromyalgia prevalence and related factors in a large registry of patients with systemic lupus erythematosus. Clin Exp Rheumatol.

[27] Iannuccelli C, Spinelli FR, Guzzo MP, Priori R, Conti F, Ceccarelli F (2012). Fatigue and widespread pain in systemic lupus erythematosus and Sjögren’s syndrome: symptoms of the inflammatory disease or associated fibromyalgia?. Clin Exp Rheumatol.

[28] Yee C, Crabtree N, Skan J, Amft N, Bowman S, Situnayake D (2005). Prevalence and predictors of fragility fractures in systemic lupus erythematosus. Ann Rheum Dis.

[29] Carli L, Tani C, Spera V, Vagelli R, Vagnani S, Mazzantini M (2016). Risk factors for osteoporosis and fragility fractures in patients with systemic lupus erythematosus. Lupus Sci Med.

[30] Abu-Shakra M, Buskila D, Shoenfeld Y (2003). Osteonecrosis in patients with SLE. Clin Rev Allergy Immunol.

[31] Huang FF, Fang R, Nguyen MH, Bryant K, Gibson KA, O’Neill SG (2020). Identifying co-morbid fibromyalgia in patients with systemic lupus erythematosus using the Multi-dimensional Health Assessment Questionnaire. Lupus.

[32] Bair MJ, Krebs EE, Fibromyalgia (2020). Ann Intern Med.

[33] Friedman AW, Tewi MB, Ahn C, McGwin G, Fessler BJ, Bastian HM (2003). Systemic lupus erythematosus in three ethnic groups: XV. Prevalence and correlates of fibromyalgia. Lupus.

[34] Torrente-Segarra V, Carbonell-Abelló J, Castro-Oreiro S, Manresa Domínguez JM (2010). Association between Fibromyalgia and psychiatric disorders in systemic lupus erythematosus. Clin Exp Rheumatol.

[35] Floris A, Piga M, Cauli A, Mathieu A (2016). Predictors of flares in systemic Lupus Erythematosus: preventive therapeutic intervention based on serial anti-dsDNA antibodies assessment. Analysis of a monocentric cohort and literature review. Autoimmun Rev.

[36] Waldheim E, Elkan A-C, Bergman S, Frostegård J, van Vollenhoven R, Henriksson EW (2013). Extent and characteristics of self-reported pain in patients with systemic lupus erythematosus. Lupus.

[37] Waldheim E, Elkan A-C, Pettersson S, van Vollenhoven R, Bergman S, Frostegård J (2013). Health-related quality of life, fatigue and mood in patients with SLE and high levels of pain compared to controls and patients with low levels of pain. Lupus.

[38] Gormezano NWS, Silva CA, Aikawa NE, Barros DL, da Silva MA, Otsuzi CI (2016). Chronic arthritis in systemic lupus erythematosus: distinct features in 336 paediatric and 1830 adult patients. Clin Rheumatol.

[39] Pettersson S, Lövgren M, Eriksson LE, Moberg C, Svenungsson E, Gunnarsson I (2012). An exploration of patient-reported symptoms in systemic lupus erythematosus and the relationship to health-related quality of life. Scand J Rheumatol.

[40] Baker K, Pope J (2009). Employment and work disability in systemic lupus erythematosus: a systematic review. Rheumatol Oxf Engl.

[41] Malcus Johnsson P, Sandqvist G, Bengtsson A, Nived O (2008). Hand function and performance of daily activities in systemic lupus erythematosus. Arthritis Rheum.

[42] Alarcón GS, McGwin G, Uribe A, Friedman AW, Roseman JM, Fessler BJ (2004). Systemic lupus erythematosus in a multiethnic lupus cohort (LUMINA). XVII. Predictors of self-reported health-related quality of life early in the disease course. Arthritis Rheum.

[43] Kiani AN, Strand V, Fang H, Jaranilla J, Petri M (2013). Predictors of self-reported health-related quality of life in systemic lupus erythematosus. Rheumatol Oxf Engl.

[44] World Medical Association (2013). World Medical Association Declaration of Helsinki: ethical principles for medical research involving human subjects. JAMA.

